# Grain size and shape fractal characteristics of gangue in the process of ’jaw breaking-ball milling’

**DOI:** 10.1371/journal.pone.0281513

**Published:** 2023-02-17

**Authors:** Wenzhe Gu, Lei Zhu, Zhicheng Liu, Zhiwei He

**Affiliations:** 1 School of Energy and Mining Engineering, China University of Mining and Technology, Beijing, China; 2 China Coal Energy Research Institute, Xi’ an, China; Universidad de Almeria, SPAIN

## Abstract

This study investigates the fractal characteristics of the particle size and shape distribution of gangue powder in the "jaw crushing-ball milling" process using mudstone gangue. For this, fractal theory, laser particle size analyzer, scanning electron microscope and other mesoscopic research methods were introduced. This study has several main factors, including the discharge port width in the jaw crushing stage, the grinding particle size, ball-to-powder ratio in the ball milling stage, and the fractal dimension changes of the gangue in different crushing stages. The results indicate that in the process of "jaw crushing-ball milling", gangue’s particle size and shape fractal dimension values changed periodically. During the jaw crushing stage, the particle size fractal dimension increases with the width of the discharge opening, ranging from 1.85 to 1.92. The value of the shape fractal dimension varies from 2.65 to 2.84. Ball milling causes the fractal dimension value of gangue particle size to increase with time before agglomeration and decrease after agglomeration. By comparing different in-grinding particle sizes and ball-to-powder ratio, it is found that the fractal dimension value of gangue particle size decreases with the increase of in-grinding particle size and increases with the increase of ball-to-powder ratio. The final gangue’s particle size fractal dimension value is concentrated between 2.5 and 2.8. The fractal dimension of particle shape increases with the increase of the grinding particle size, and decreases with the increase of ball-to-powder ratio. A ball-to-powder ratio greater than 6 gradually reduces its influence on fractal dimensions, and the final shape dimension lies between 1.06 and 1.16. In addition, the increase/decrease range of particle size and shape fractal dimension decreases with the increase of ball milling time, which is also consistent with the grinding kinetics theory. As a result of the changes in particle size and shape fractal dimensions, parameters such as jaw crusher discharge port width, grinding particle size, and ball-to-powder ratio are calculated to provide a theoretical basis for the entire crushing process in the "jaw crusher-ball milling" crushing process.

## 1 Introduction

In recent years, the large-scale mining of coal resources has resulted in producing and accumulating a large number of coal gangue on the surface. This phenomenon widely exists in major coal mines. Currently, dealing with mine gangue and efficiently utilizing it has become a major engineering and scientific concern. Chen Y et al have successively pointed out the adverse effects of long-term accumulation of gangue in coal mines on the environment, such as land, rivers, and air [1, 2]. Then scholars from various countries have proposed multiple ways of gangue resource utilization [2], such as applying gangue to building materials [3], production of cement [4], geotechnical materials [5] and so on. Based on the above research results, it can be deduced that although the efficient utilization of coal gangue has received attention from experts and scholars in different fields[6–8], efficient utilization requires crushing the coal gangue from its surface first, and this process can only be carried out once the particle shape and size meet the required size. This requires the gangue’s on-site realization from the surface’s bulk size to the small particle size of the multi-path application stage. However, few reports are documented that explain the crushing of coal gangue under the action of the crushing device.

Small block groups dominate the macroscopic fragmentation of gangue under the action of mechanical power, and the small blocks are composed of smaller crack evolution and aggregation. This similar behavior leads to self-similarity in the process of breaking blocks. As a powerful tool to describe nonlinear self-similar systems, the fractal theory has been widely used in particle size and shape characterization of powders [9–14]. Hu L [15] proposed the concept of abstract fractal features of damage parameters and applied it to the crushing stage of rocks. The relationship between fractal damage and the number of fragments was established. Based on fractal theory, Zhou et al. [16] analyzed the fractal characteristics of granite under dynamic impact crushing and proposed a crushing model based on renormalization by analyzing the correlation between fractal dimension and energy density. Maria et al. [17] proposed a new method for quantitative analysis of volcanic particle shape based on fractal geometry. To reveal the complexity and variability of volcanic particle morphology, Yuan et al. [18] calculated the surface fractal dimension using the slit island and the box-counting method and utilized the contour and surface fractal dimension to describe the TiN coating surface to characterize the degree of surface wear.

The above studies using fractal theory have expounded the important role in particle size and shape and demonstrated the fractal theory’s feasibility in characterizing particle size and shape. Still, most focus on theoretical aspects or non-dynamic crushing fields, and none have clarified the fractal dimension. The crushing of gangue in mine site often requires multi-stage crushing to meet the expected requirements. It is necessary to study the particle size and shape fractal dimension of each link in the crushing process. In addition, there are many ways of recycling gangue, which have different requirements on the grain size and shape of gangue. It will be of great significance for the recycling utilization of gangue if the fractal dimension of the grain size and shape of gangue could be controlled to meet the requirements of recycling utilization of gangue by controlling the crushing process parameters. Thus, exploring the change of the fractal dimension of the particle size and shape of gangue in the process of "jaw breaking—ball milling" provides a theoretical basis for the parameter selection of the whole crushing process, and helps to achieve accurate control of the particle size and shape of gangue.

## 2 Experimental methods

### 2.1 Preparation of gangue samples

**Fig 1:**
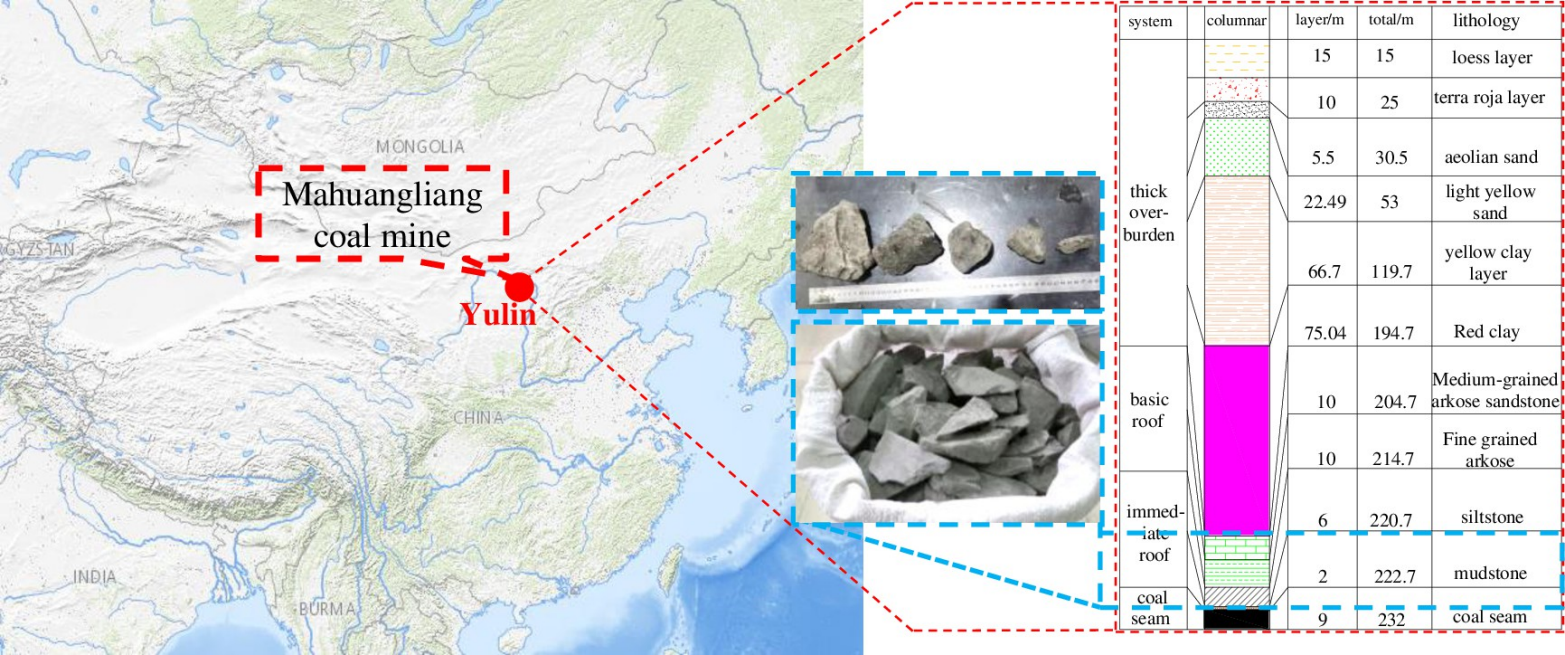
Schematic diagram of gangue sampling site and stratum.

The gangue samples used in this study were taken from the surface filling station of Mayhuangliang Coal Mine in Yulin, Shanxi Province, as shown in Fig 1. The gangue accumulated on the surface is generally irregular in shape, with sharp edges and corners, and is not smooth. Its lithology and mechanical properties are shown in Table 1 [19].

**Table 1:** Mechanical parameters of coal gangue samples.

Sample	Lithology	Elastic Modulus(GPa)	Density (kg/m^3^)	Poisson’s ratio	Compressive Strength(MPa)	Cohesion (MPa)	Internal friction angle/ °
Gangue	mudstone	4.64	1430	0.15	13.66	3.01	41.94

**Fig 2:**
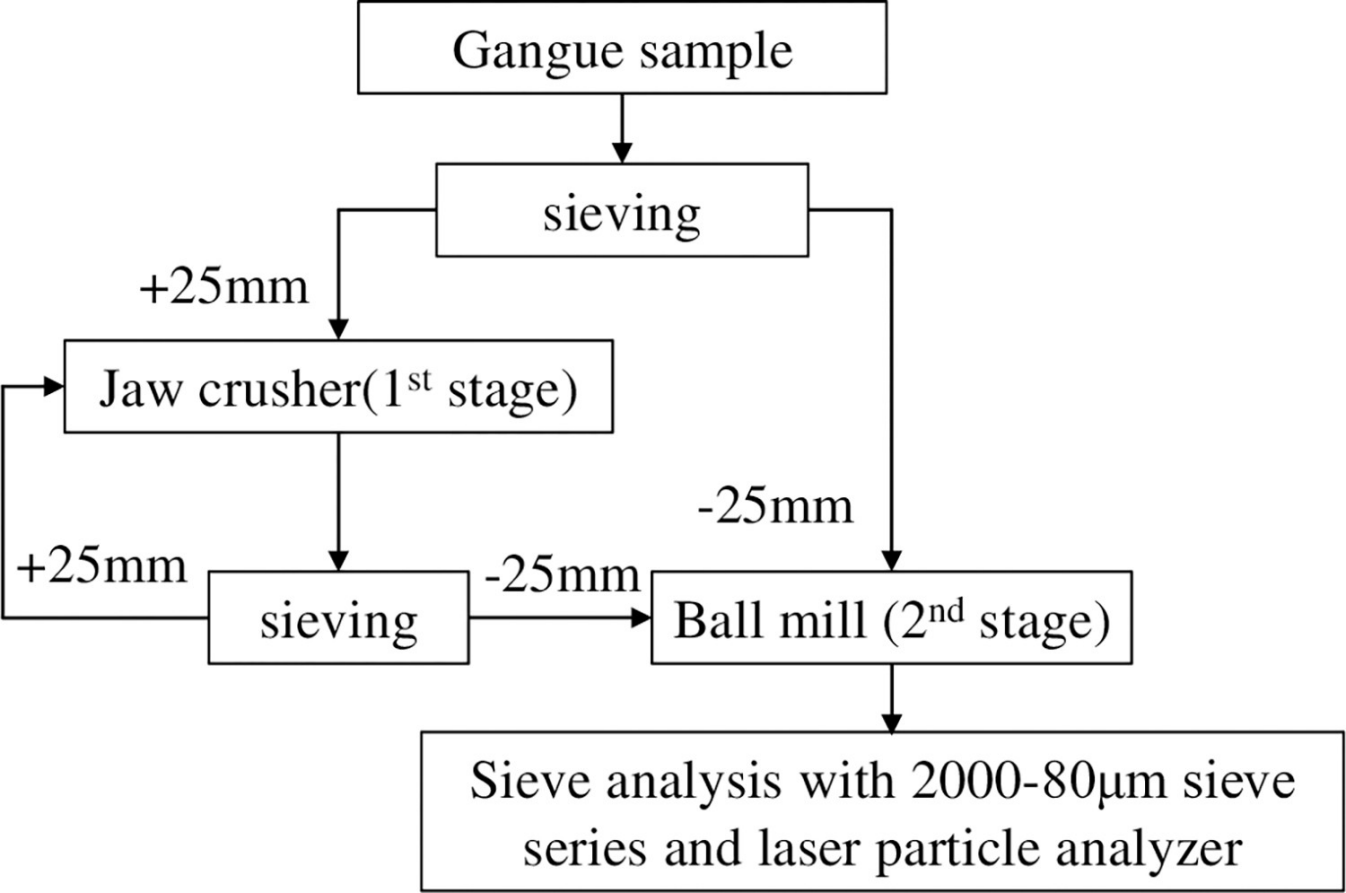
On-site gangue crushing flow chart.

According to the on-site investigation, it is currently planned to perform "jaw crushing-ball milling" treatment on the surface gangue to meet the purpose of multi-resource utilization of gangue. The specific process is shown in Fig 2. To optimize the on-site crushing process and crushing efficiency, gangue particle size and shape changes were studied under different crushing stages and influencing factors.

### 2.2 Test apparatus and plan

#### 2.2.1 Crushing apparatus

In this study, the original gangue was taken as the object. The gangue was subjected to jaw crushing and ball milling tests to explore the influence of various factors on the fractal dimension in the crushing process. The required crushing equipment mainly includes:

(1) Jaw Crusher

**Fig 3:**
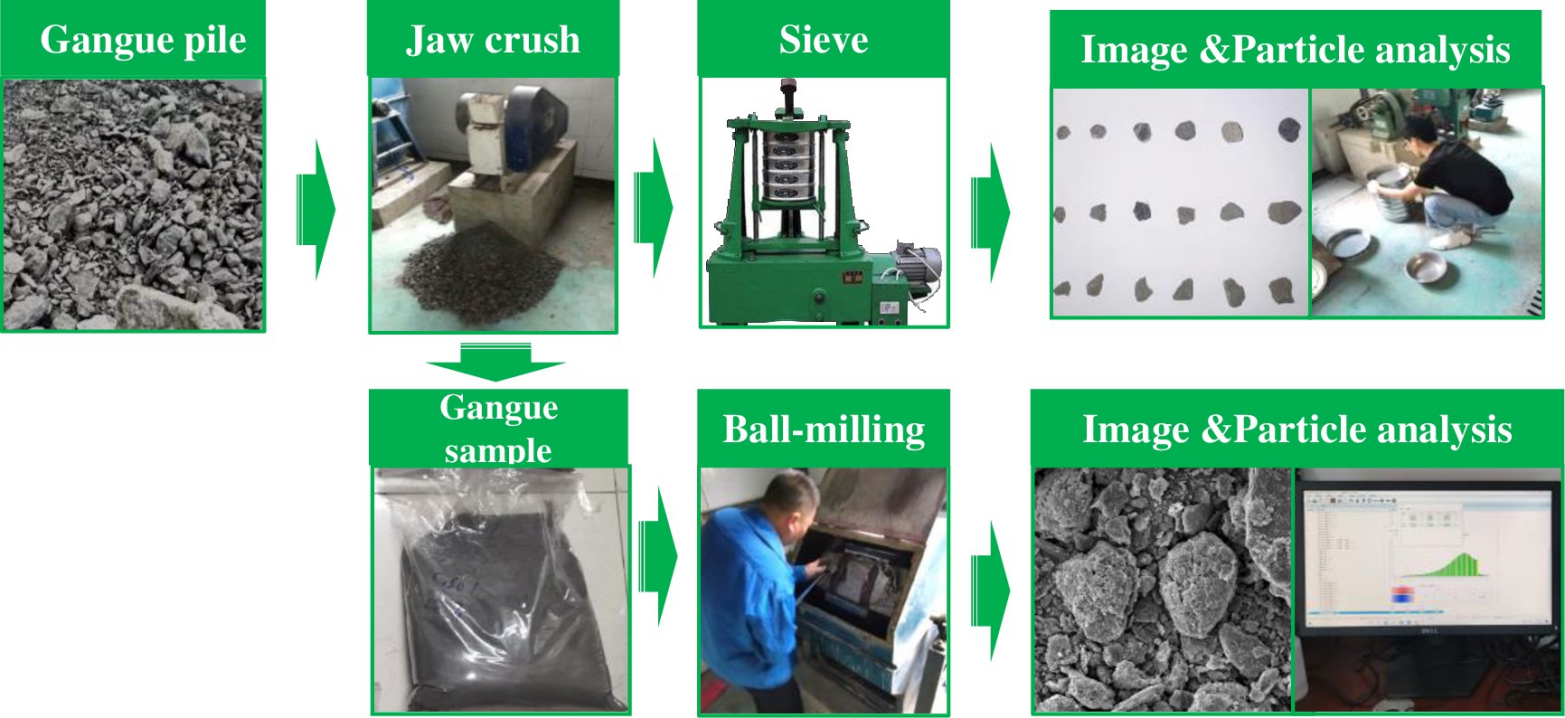
Fractal dimension test of particle image and particle size during the "jaw crushing-ball milling" process of gangue in the laboratory.

According to the crushing process on-site, the jaw crusher model used in the indoor crushing experiment is PEF60×100, the maximum feeding particle size is 48 mm, and the power is 1.5 kw (Fig 3). The width of the discharge port is 6~10 mm. The equipment can obtain gangue products with different particle size gradations by adjusting the discharge port width.

(2) Ball Mill

The model of the ball mill used in this test is SMφ500×500mm test mill (Fig 3), the grinding medium is steel balls, the maximum single charge is 5 Kg, the maximum load of the grinding body is 100 Kg, the power is 1.5 kw, and the speed is 48 rpm. Rolling bearings mainly support the working principle, and the cylinder is driven to rotate through the transmission device so that the material and steel balls rotate together with the cylinder. The dropping, impact, impact and self-grinding operations are completed in the cylinder to achieve material grinding [20].

#### 2.2.2 Test procedure

Fig 3 shows the test steps for particle size and shape fractal dimension testing of gangue in the laboratory "jaw breaking-ball milling" process:

Step 1: The gangue retrieved from the site is cleaned and dried, and then jaw crushing operations are carried out in sequence with different discharge opening widths. The crushed gangue particles are sieved on a vibrating screen for 10 minutes, and the quality of the gangue particles of different fractions is weighed. At the same time, pictures of gangue of different grades were acquired (Fig 3) to provide data for analyzing the shape fractal dimension of gangue.

Step 2: Start the ball mill device; debug the test ball mill to test whether it is running normally, and then according to the ball mill crushing experiment plan table (Table 2), weigh the designed grinding medium and gangue of the corresponding quality using an electronic balance, mix them evenly, and fill the barrel with them.

**Table 2:** Ball mill crushing experiment scheme.

Grinding particle sizes	Material weight /kg	Ball weight /kg	Mass ratio of ball to gangue	Grinding time /min
6mm	8.34	50	6	10~60
8mm				
10mm				
6mm	16.67	50	3	
	8.34	50	6	
	5.55	50	9	
	4.16	50	12	
	3.33	50	15	

Step 3: Start the ball mill, stop working every 10 minutes after the test ball mill runs, open the sealing plate on the end face of the cylinder, evenly divide the gangue particles with a sample divider, sieve the gangue particles for 10 minutes into the vibrating sieve, and pass the sample through a laser particle size analyzer and an electronic scale.The particle size distribution and morphology were measured by microscopy (Fig 3).

Step 4: Reseal the end face of the ball mill barrel, then start the test ball mill to run for 10 min, 20min, 30min, 40min, 50min, and 60min in sequence, and repeat step 3.

Step5: According to the test plan in Table 2, change the grinding particle size and the ratio of ball to gangue, and repeat steps 2 to 4.

## 3 Method and theory

### 3.1 Particle size analysis method

The particle size ratio of the gangue after jaw crushing is based on the quality of each particle grade after sieving, while the particle size of the gangue in the ball milling process is measured using a BT-9300ST laser particle sizer. Fractal analysis is carried out on the gangue samples in the above crushing process using fractal theory. The specific process is as follows:

According to the basic definition of fractal [21], the number N(r) of particles smaller than *r* satisfies

$$N(r)\propto r^{-D}$$ (1)

In the formula, *D* is the fractal dimension of the particle distribution.

After the gangue is crushed, it is often difficult to calculate the number of particles, but the mass of each particle can be obtained by sieving and weighing. Assuming that the mass and density of the particles are the same, Tyler et al. [22, 23] The relationship between particle sizes is as follows:

$$\frac{M(d<d_{i})}{M_{t}}={\left(\frac{d_{i}}{d_{\max}}\right)}^{3-D}$$ (2)

In the formula, *M(d<d*_*i*_*)* is the mass of particles whose particle size *d* is smaller than the particle size *d*_*i*_, *M*_*t*_ is the total mass of the sample, and *d*_*max*_ is the maximum particle size of the particles. Taking the logarithm of both sides of Eq (2), we can get:

$$\log[\frac{M(d<d_{i})}{M_{t}}]=(3-D)\log(\frac{d_{i}}{d_{\max}})$$ (3)

Formula(3) shows that there is a linear relationship between *lg*[*M*(*d*<*d*_*i*_)/*M*_*t*_] and *lg*[(*d*_*i*_/*d*_*max*_)], and its slope is *(3-D)*.

### 3.2 Image processing method

**Fig 4:**
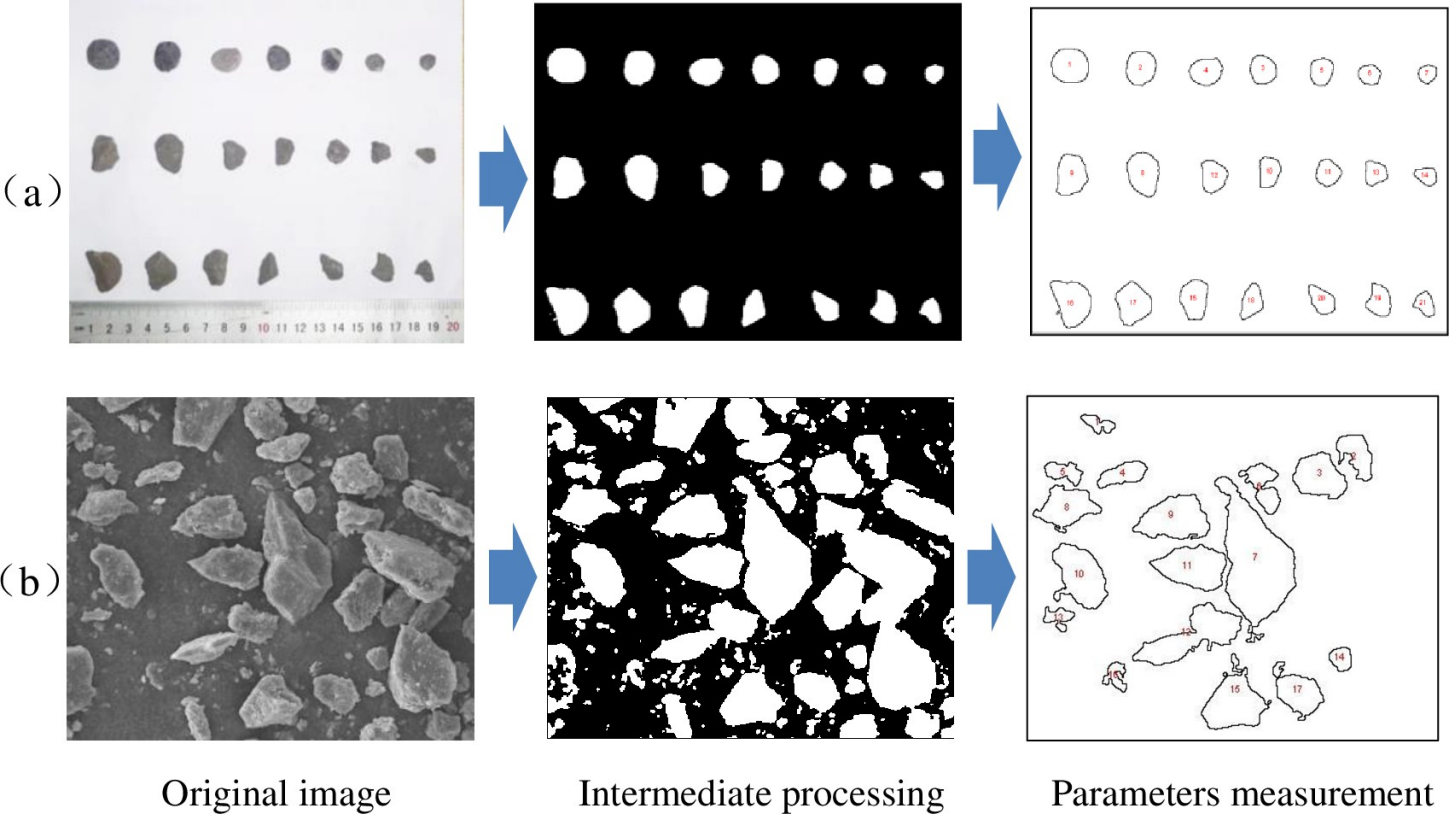
The quantification process of the morphological parameters of the gangue material.

For the large-grained gangue after jaw crushing, the image of the gangue particles is obtained through camera photography because the particles are large, and the edges and corners are obvious. For the observation of the external morphology of the gangue particles after ball milling, the JSM-IT500 scanning electron microscope was used to obtain ESEM pictures. Then, the camera and ESEM pictures are used to obtain parameters such as the area and perimeter of the gangue using digital graphic analysis and processing software such as Adobe Photoshop and Sigma Scan pro. The quantification process of the gangue structure and morphology is shown in Fig 4.

In this paper, the area perimeter method (C-S method) is used to analyze the fractal dimension of the particle shape. This method is not only suitable for the image processing of large particles of gangue after jaw crushing but also suitable for the image processing of tiny particles of gangue observed under the electron microscope [24]. After Mandelbrot analyzed the C-S relationship in regular geometric figures, he gave the C-S relationship in fractals as [25]:

$$\ln S=A+\frac{D}{2}\ln C$$ (4)

In the formula, *S* is the perimeter of the particle; *C* is the area of the particle; *D* is the shape fractal dimension of the particle.

After drawing a double logarithmic lnC-1nS diagram of the area and perimeter of gangue particles, the linear slope D/2 was obtained by linear fitting, and the 2 times of the slope represented the fractal dimension of the particles’ outer contours. Different particle fractal dimension values *d*_1_, *d*_2_,… *d*_n_ measured multiple times by the above method are taken as the arithmetic mean.

## 4 Test results and discussion

### 4.1 Fractal dimension characterization of particle size and shape of gangue during jaw crushing

**Fig 5:**
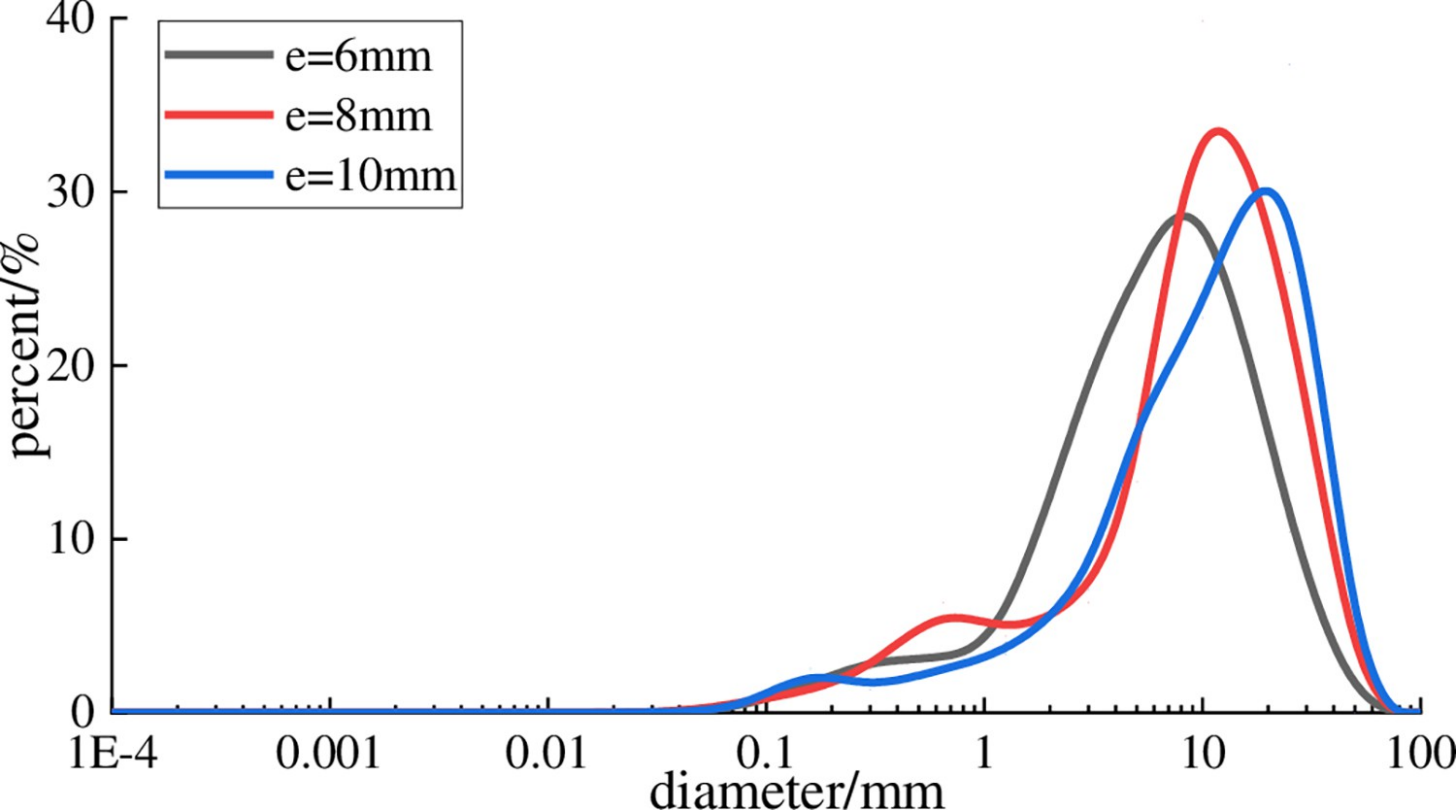
Grain size distribution of gangue with different width of ore outlet.

Table 3 describes the particle size of the three types of ore discharge opening widths for statistical analysis, and each broken sample is counted as e-6, e-8, and e-10. The particle size distribution curve drawn according to Table 3 is shown in Fig 5. As the width of the discharge opening increases, the particle size distribution trend of the gangue remains the same under the width of each discharge opening.

**Table 3:** Sieving test results of gangue fragment with different width of ore outlet.

Gangue sample	The cumulative mass percentage at a specific mesh size(%)							
	0.3mm	0.6mm	1.2mm	2.5mm	5mm	10mm	25mm	d_mean_/mm
e-6	5.08	3.08	4.08	19.71	35.33	27.35	5.37	6.42
e-8	4.43	2.33	4.43	10.33	33.29	29.87	15.32	8.80
e-10	2.21	2.15	2.38	6.1	30.72	32.14	24.3	11.13

**Fig 6:**
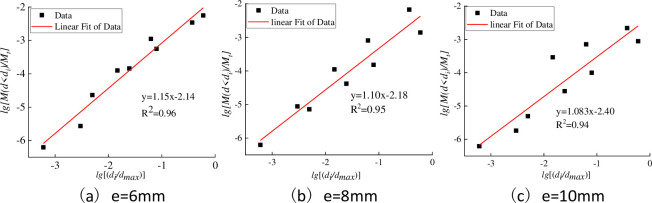
Comparison of fractal Model (solid line) with experimental data (symbol), X = *lg*[*(d*_*i*_*/d*_*max*_*)*] and Y = *lg*[*M(d<d*_*i*_*)*/*M*_*t*_]. (a) e = 6mm. (b) e = 8mm. (c) e = 10mm.

According to the calculation method of particle size fractal dimension, fractal analysis was carried out on the gangue produced by each row of mines in turn, and the results are shown in Fig 6. Under the double logarithmic coordinate, the *lg*[*M(d<d*_*i*_*)*/*M*_*t*_]- *lg*[*(d*_*i*_*/d*_*max*_*)*] distribution function of the gangue under different discharge opening widths is in good line with the linear relationship [26, 27]. The regression curves of the scatter plots under any width of the discharge opening are all straight lines, and the *R*^2^ of the regression coefficient is at least 0.98, indicating that the gangue has an obvious fractal characteristic in the jaw crushing process. The fractal dimension values of the gangue under the three discharge openings are 1.85, 1.90, and 1.917 in turn. As the width of the discharge opening increases, the fractal dimension value of the gangue particle size shows an increasing trend, but the fractal dimension value is only in the range of 1.85~ 1.92. Combining with Table 3, it can be seen that although the fractal dimension of particle size of gangue under the width of the discharge opening is small, the difference in particle size structure is relatively large. At the same time, after sieving by standard sieve, the gangue in each particle class is selected for particle shape dimension analysis. The fractal dimension statistics of all particle sizes after image processing are shown in Table 4. Comparing the fractal dimension of the shape of gangue under different widths of the discharge opening, it can be seen that the value of the fractal shape of the gangue only varies from 2.65 to 2.84, and its value has little correlation with the width of the discharge opening.

**Table 4:** Calculated particle size fractal dimension and shape fractal dimension.

Gangue sample	Average size (mm)	Fractal dimension *D*	Correlation coefficient *R*^2^	Fractal dimension of shape
e-6	6.42	1.85	0.99	2.84
e-8	8.80	1.90	0.98	2.71
e-10	11.13	1.917	0.98	2.80

### 4.2 Fractal dimensional characterization of different grinding particle sizes

It can be seen from the above analysis that the overall particle size structure of the gangue produced from different discharge opening widths is quite different. To investigate the changes in particle size and shape fractal dimension in the crushing process of "jaw crusher-ball milling", the gangue produced by different discharge opening widths were ball-milled respectively, and the influence of the in-grinding particle size on the fractal dimension was investigated.

#### 4.2.1 Fractal dimension characterization of particle size

**Fig 7:**
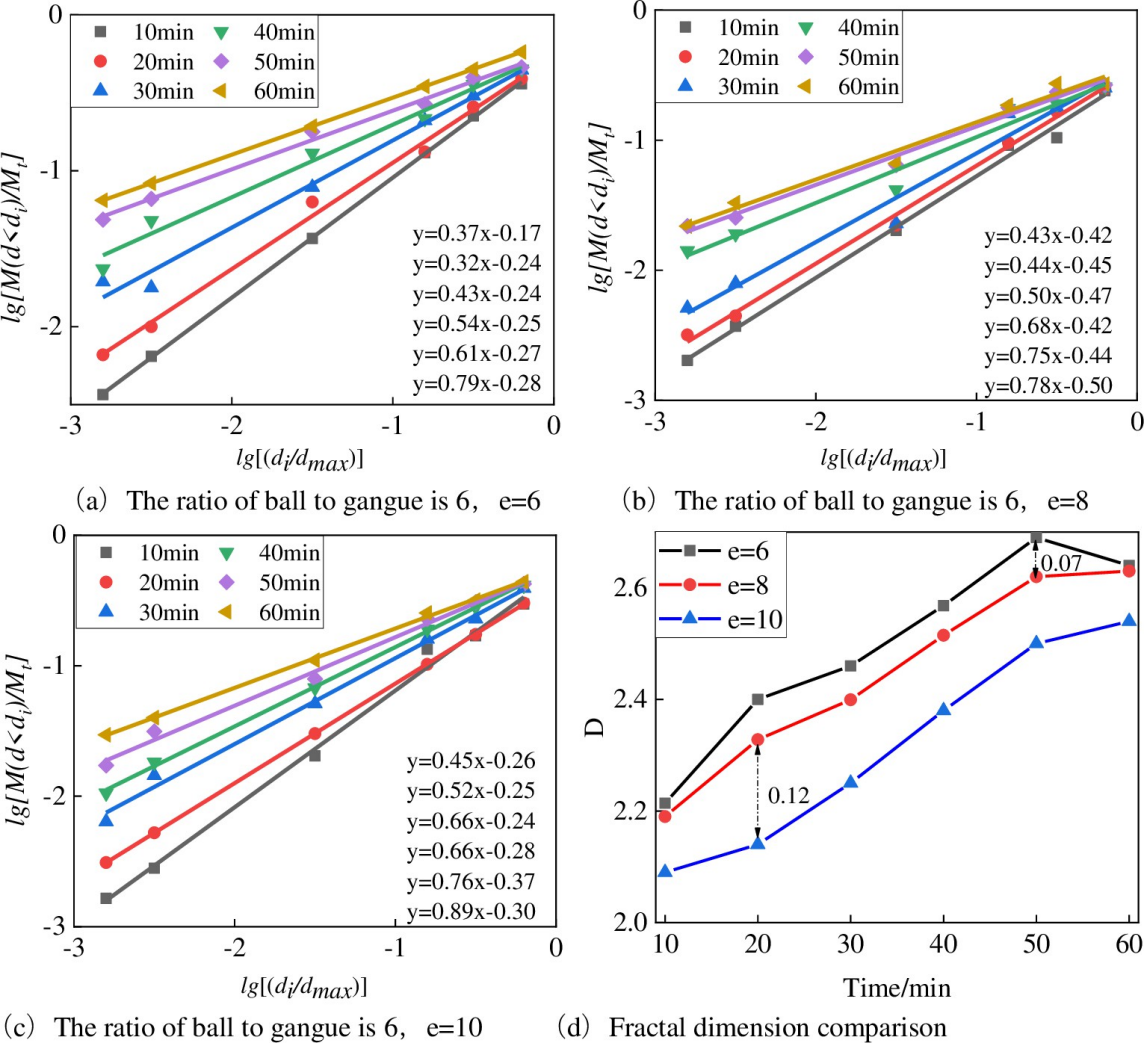
Changes of particle size fractal dimension of gangue with different grinding particle sizes during ball milling. (a) The ratio of ball to gangue is 6, e = 6. (b) The ratio of ball to gangue is 6, e = 8. (c) The ratio of ball to gangue is 6, e = 10. (d) Fractal dimension comparison.

Fig 7 depicts the change of the size fractal dimension of the gangue with different grinding particle sizes during the ball milling process. Fig 7(A)–7(C) shows that the gangue with different grinding particle sizes has obvious fractal characteristics during the ball milling process. And the fractal dimension of each particle size of gangue increases with time. The difference is that the fractal dimension of e-8 and e-10 samples increase continuously with the ball milling time, e-6 increases in the range of 10-50min, and decreases in the range of 50-60min. The fractal dimension decreases with time due to gangue agglomeration in the ball milling process. The distribution fractal dimension reflects the uniformity of particle distribution. The larger the fractal dimension is, the easier the material is to be pulverized and the better the particle distribution concentration is. The fractal dimension of the gangue of three incoming particle sizes follows an order of e-6>e-8>e-10 based on comparison of the gangue of three incoming particle sizes. The maximum difference between the fractal dimension values of e-6 and e-8 is only 0.07, while the difference between e-8 and e-10 is larger, and the maximum difference value is 0.12. This means that the influence of the width of the discharge port on the fractal dimension value in the ball milling stage increases with the increase of the width of the discharge port. The smaller the gangue particle size is, the easier it is to pulverize the gangue during the ball milling process, and the better the concentration is.

#### 4.2.2 Fractal dimension representation of shape

**Fig 8:**
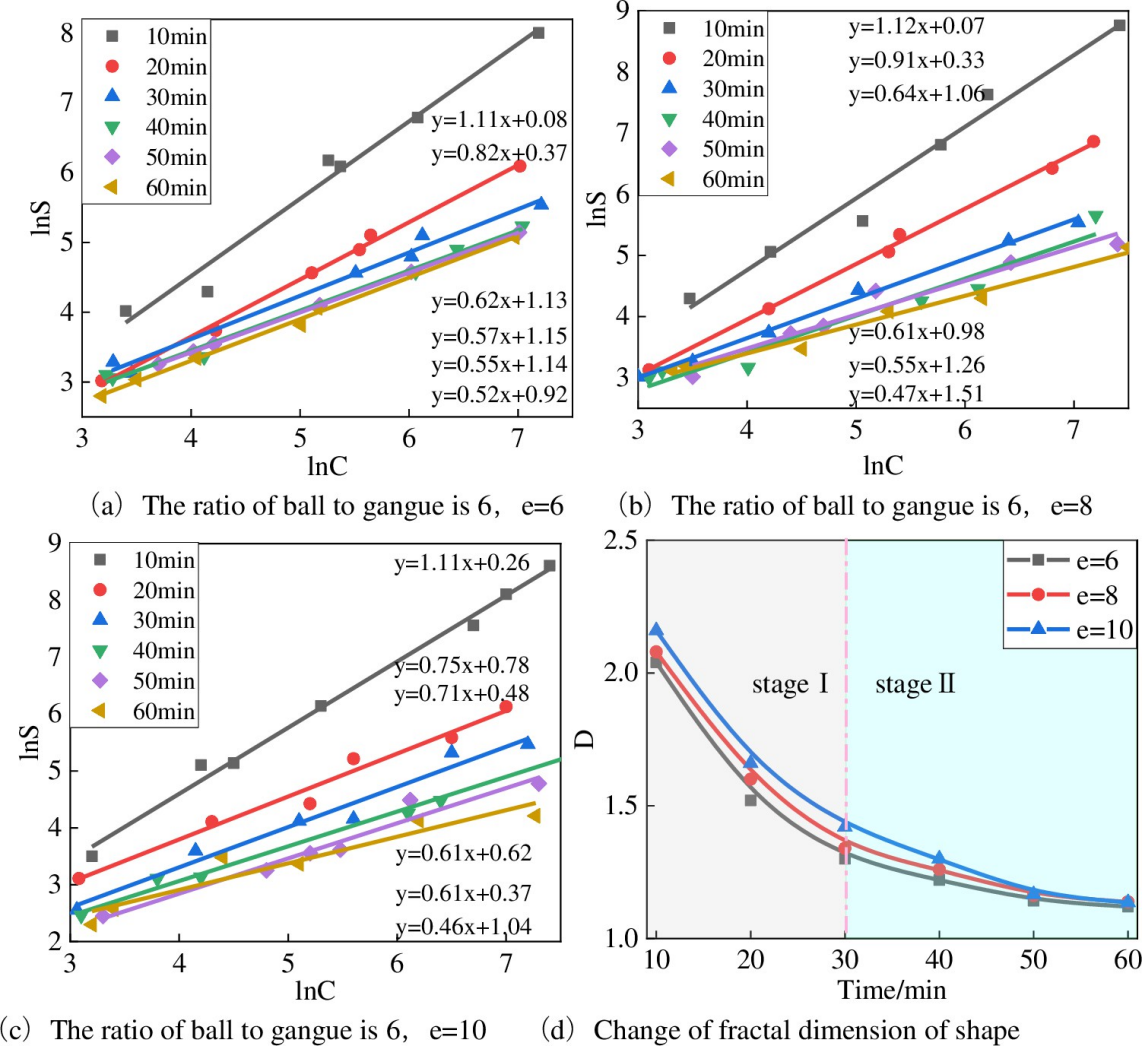
The shape fractal dimension change of gangue produced by different discharge openings during the ball milling process. (a) The ratio of ball to gangue is 6, e = 6. (b) The ratio of ball to gangue is 6, e = 8. (c) The ratio of ball to gangue is 6, e = 10. (d) Change of fractal dimension of shape.

The fractal dimension of the shape reflects the regularity of the particle shape. The larger the fractal dimension, the more edges and corners the particle contains, and the more irregular the shape is [28–30]. Fig 8 shows the shape and fractal dimension changes of gangue with different grinding particle sizes during the ball milling process under the gangue ratio of 6 (m = 6). The particle shapes of the gangue with different grinding particle sizes have obvious fractal characteristics during the ball milling process. As shown in the figure, the fractal dimension value of particle shape decreases with time, indicating that particles become spherical and elliptical in shape with increasing ball milling time. Comparing the 0 value of the shape fractal dimension of the gangue with the three feed sizes in the ball milling process, it can be found that the fractal dimension value of the shape changes in stages in time (Fig 8(D)). During the ball milling process, the fractal dimension of the gangue shape decreased rapidly in stage I, and the decreasing rate gradually slowed down in stage II. The order of the fractal dimension of the shape at any time point is e-6 < e-8 < e-10, indicating that reducing the grinding particle size can significantly reduce the fractal dimension of the particle shape during the ball milling process.

**Fig 9:**
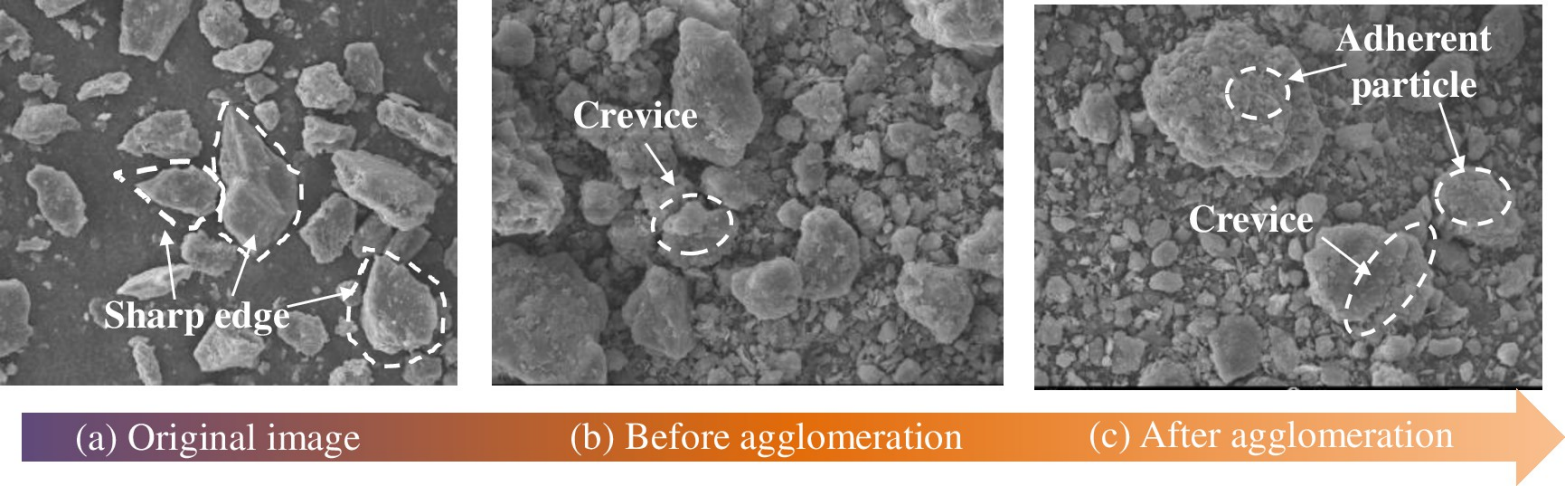
Electron microscope images of gangue at different ball milling stages. (a) Original image. (b) Before agglomeration. (c) After agglomeration.

Combining with Fig 9, it is observed that the shape fractal dimension of the particles does not change as the gangue of e-6 agglomerates [31, 32] at 50 min but continues to decrease slowly. The reason for this can be evident from the morphology change of the gangue under the electron microscope. The initial gangue has sharp edges and corners, and the shape fractal dimension value is large. There is more wear, smooth surfaces, and mostly ellipsoidal or spherical shapes. With the improvement of the shape degree of the gangue particles, it can be seen that the fractal dimension of the gangue gradually decreases before the agglomeration time. It can be demonstrated from the electron microscope image that the surface of the gangue is covered with scaly or granular particles after agglomeration, and the overall roughness increases, but the shape of the gangue is still ellipsoid, showing that the fractal dimension decreases in a small range.

### 4.3 Fractal dimension characterization under different ball-to-powder ratios

#### 4.3.1 Fractal dimension characterization of particle size

**Fig 10:**
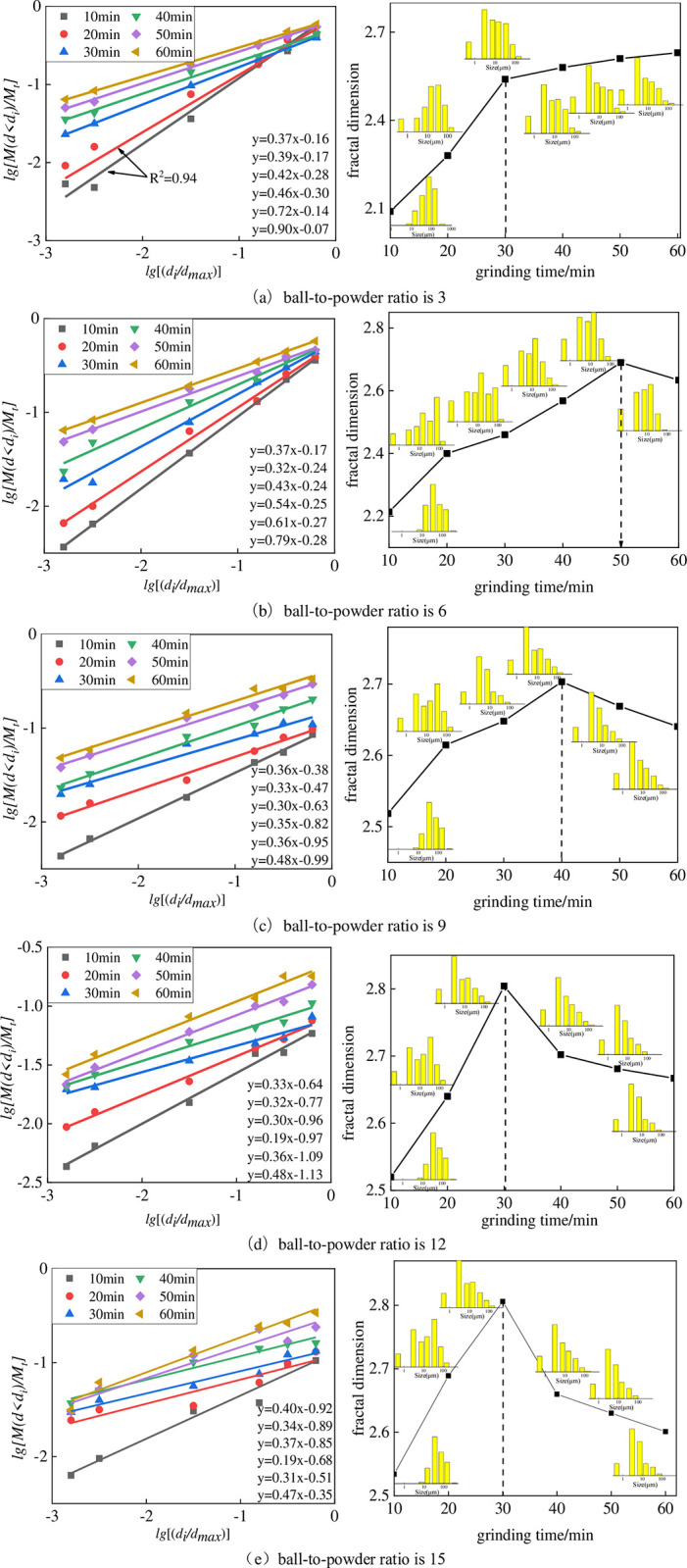
Fractal characteristics of gangue particle size distribution under different ball-to-powder ratios. (a) ball-to-powder ratio is 3. (b) ball-to-powder ratio is 6. (c) ball-to-powder ratio is 9. (d) ball-to-powder ratio is 12. (e) ball-to-powder ratio is 15.

Fig 10 depicts the fractal characteristics of the particle size distribution of gangue under the conditions of different ball-to-powder ratios. When ball-to-powder ratio is 3, the fractal dimension of the gangue particle size increases with time. Firstly, when the ball milling time is short (10~20 min), the fractal characteristics are weakened, resulting in a correlation coefficient *R*^*2*^ of only 0.94 after fitting the slope b; the linear characteristics become more evident after the ball milling time exceeds 20 minutes, and the correlation coefficient R^2^ after fitting reaches 0.98. The above analysis shows obvious fractal characteristics. Secondly, the growth rate of fractal dimension before 20 min is significantly greater than that after 20 min, which indicates that the change of gangue grain size and structure weakens with time. Under these conditions, when the number of grinding media is small and the ball milling time is short, part of the gangue does not contact the grinding media, so the gangue crushing is insufficient, and the overall fitting is not good. On the other hand, according to the grinding kinetic theory [33–36], as the particle size decreases, the energy required for further grinding will gradually increase, making it more challenging to break the fine particles further. At the same time, due to the buffer formed by the generated fine powder in the ball mill, the crushing of coarse-grained gangue is hindered, and the ball milling efficiency is gradually reduced, so the increasing trend of fractal dimension in the figure is slowed down [37].

When ball-to-powder ratio is greater than 3, it can be seen from Fig 10(B)–10(D) that there is a time node for the fractal dimension value of the gangue with the increase of the ball milling time so that the fractal dimension value of the gangue has a maximum value. If the ball milling time continues to increase, the fractal dimension value of the particles tends to decrease. This indicates that particles agglomerated during ball milling after this time point, resulting in a less concentrated particle distribution. In the case of a ball-to-powder ratio of 6, the extreme value of the fractal dimension is 2.69 (t = 50min). When ball-to-powder ratio is 9, the maximum fractal dimension is 2.7032 (*t* = 40min); when ball-to-powder ratio is 12, the maximum fractal dimension is 2.804 (*t* = 30min). A ball-to-powder ratio of 15 results in a maximum fractal dimension of 2.806 (t = 30 minutes). The fractal dimension value of the gangue particle size varies between 2.5 and 2.8 when ball-to-powder ratio is greater than 6, and the change trend of the fractal dimension before and after the agglomeration phenomenon is in the opposite direction.

The change of fractal dimension also reflects the change of grain size structure. The relationship between particle size distribution and fractal dimension in Fig 10 shows that with the increase of fractal dimension, the concentration of particle distribution increases: The proportion of large particles and intermediate particles decreased, and the content of small particles continued to grow.

#### 4.3.2 Fractal dimension representation of shape

**Fig 11:**
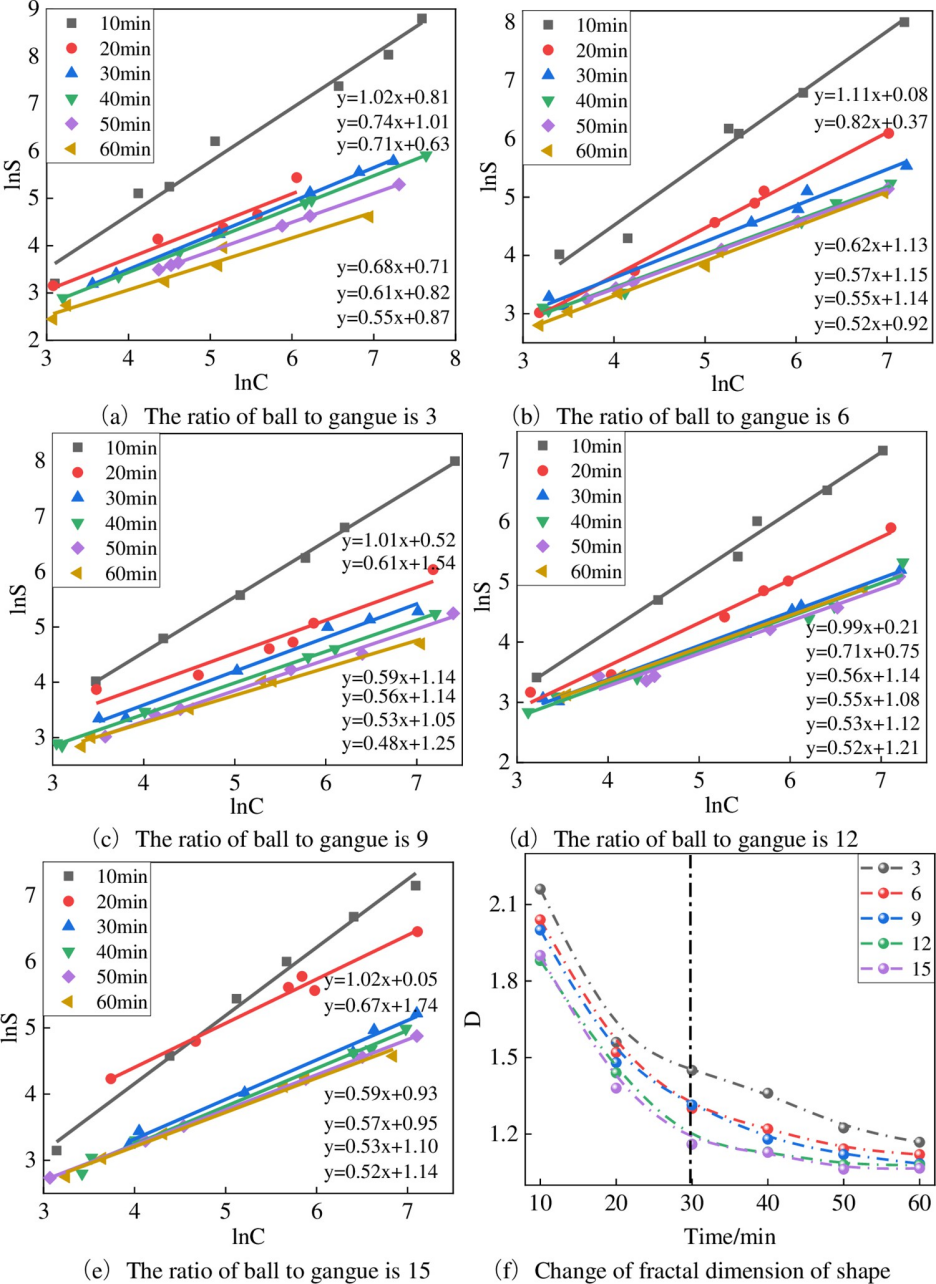
The shape fractal dimension change of gangue under different ball-to-powder ratios. (a) The ratio of ball to gangue is 3. (b) The ratio of ball to gangue is 6. (c) The ratio of ball to gangue is 9. (d) The ratio of ball to gangue is 12. (e) The ratio of ball to gangue is 15. (f) Change of fractal dimension of shape.

Fig 11 shows the fractal dimension of the shape of the gangue under the conditions of different ball material ratios. The fractal dimension value of the particle shape under the conditions of various ball material ratios in the figure shows a decreasing trend with time, indicating that the shape of the particles gradually develops to spherical and ellipsoidal with the increase of the ball milling time [38–40]. With increasing ball milling time, the shape fractal dimension of the gangue almost gradually decreases, as shown in Fig 11(F) and Table 5. A prominent periodic change is observed in the shape fractal dimension of the gangue over time: in 10 minutes, the shape fractal dimension reduces substantially. After 30 minutes, the shape fractal dimension increased with time, the decrease was very small, and the final shape dimension ranged between 1.06 and 1.16. Especially when ball-to-powder ratio is greater than 6, the gangue’s shape and fractal dimension difference during the ball milling process is small.

**Table 5:** Fractal dimension of shape under different conditions of ball-to-powder ratios in ball milling.

Time ball-to-powder ratios	10min	20min	30min	40min	50min	60min	range
3	2.16	1.56	1.45	1.36	1.224	1.168	1.16–2.16
6	2.04	1.52	1.3	1.22	1.142	1.12	1.12–2.04
9	2	1.48	1.314	1.18	1.12	1.084	1.08–2.00
12	1.88	1.44	1.16	1.13	1.08	1.078	1.07–1.88
15	1.9	1.38	1.16	1.128	1.062	1.066	1.066–1.9

The analysis shows that increasing ball-to-powder ratio by reducing the mass of the gangue in the grinding cavity essentially increases the probability of the gangue colliding per unit time, resulting in the continuous advance of the agglomeration time. However, once ball-to-powder ratio exceeds a certain value, the effect of ball-to-powder ratio on the particle size change of gangue ball milling becomes smaller and smaller, and even the increase in the number of grinding media will lead to an increase in energy consumption and aggravation of agglomeration during the grinding process.

In the grinding industry, grinding control balances reducing particle size and minimizing over-grinding to maximize grinding efficiency. Suppose the particle size of the product is too coarse or the shape is too irregular. In that case, it will bring difficulties to downstream resource utilization, resulting in increased energy consumption or reduced mineral utilization efficiency [41–43]. According to the above analysis, considering the impact of grinding particle size and ball material ratio from the perspective of the particle size and shape of the gangue, in the "jaw crusher-ball milling" crushing process, the grinding particle size of the gangue in the ball milling stage should be from the gangue with a width of 6mm or 8mm, and the ball material ratio should range between 6–9. Considering that after the agglomeration phenomenon, the fractal dimension of gangue particle size decreases, and the shape dimension decreases slowly at a very slow speed. Obviously, on the premise of taking into account the energy consumption of the ball mill and the wear of the grinding medium, we also suggest that the ball mill should be terminated before the agglomeration phenomenon occurs.

### 4.4 Changes in shape and particle size fractal dimension in "jaw crushing-ball milling" stage

**Fig 12:**
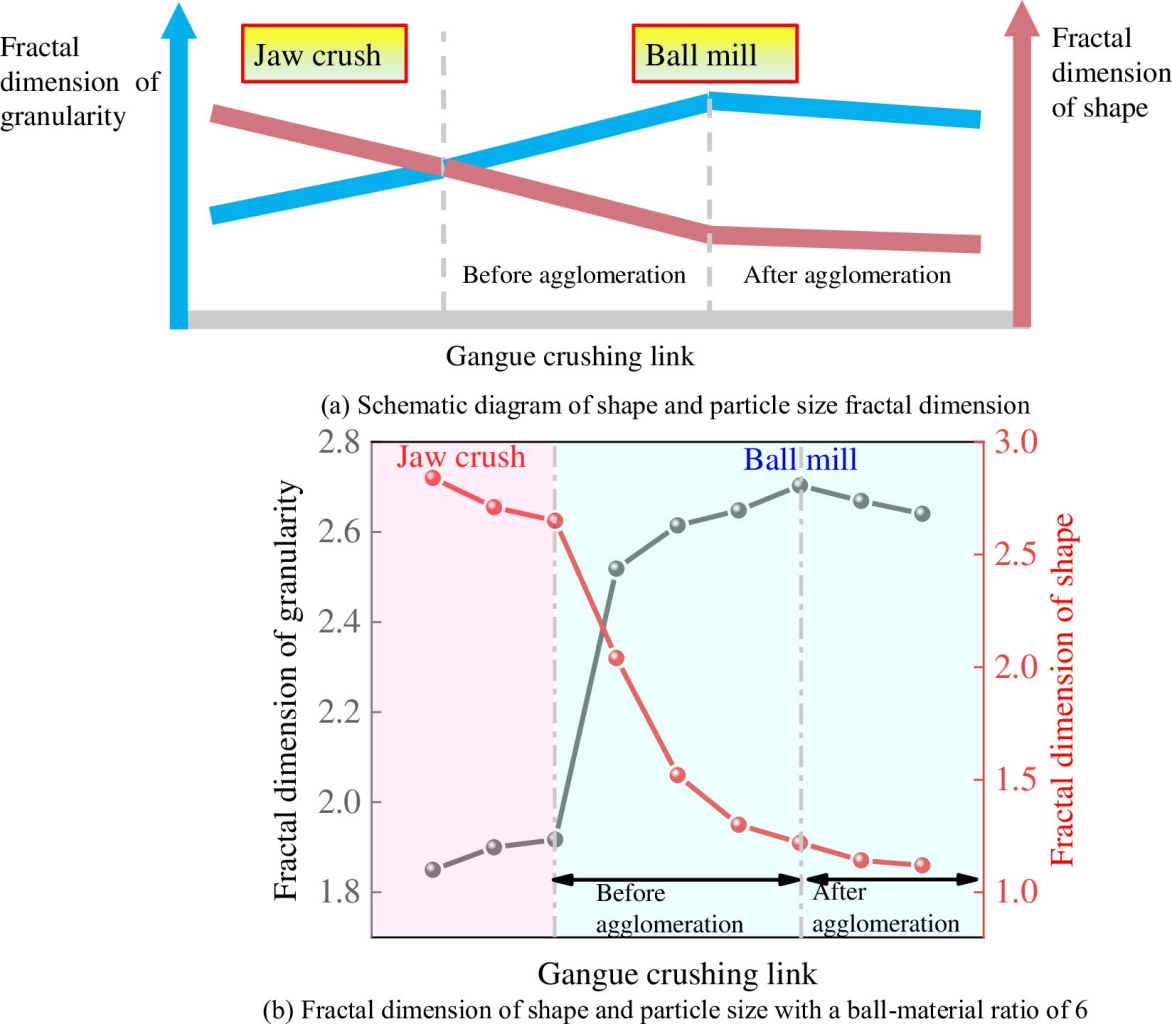
The shape and particle size fractal dimension change of gangue in the stage of "jaw crushing-ball milling". (a) Schematic diagram of shape and particle size fractal dimension. (b) Fractal dimension of shape and particle size with a ball-material ratio of 6.

Fig 12 shows the shape and size fractal dimension changes of the gangue in the "jaw crushing-ball milling" stage—taking the working condition of ball-to-powder ratio of 6 as an example. Overall, the relationship between the particle size and shape fractal dimension also lies in stages. In the jaw-crushing stage, there is almost no correlation between the gangue particle size and shape fractal dimension. In the ball milling stage, before the agglomeration phenomenon occurs, there is a positive correlation between the particle size and the shape fractal dimension of the gangue. The corresponding shape fractal dimension and particle size fractal dimension are the same before the agglomeration phenomenon occurs. After the agglomeration of gangue occurs, the fractal dimension relationship between shape and particle size is "reversed". Based on statistics, it has been found that the correlation between particle size and shape changes first, and then separates.

**Fig 13:**
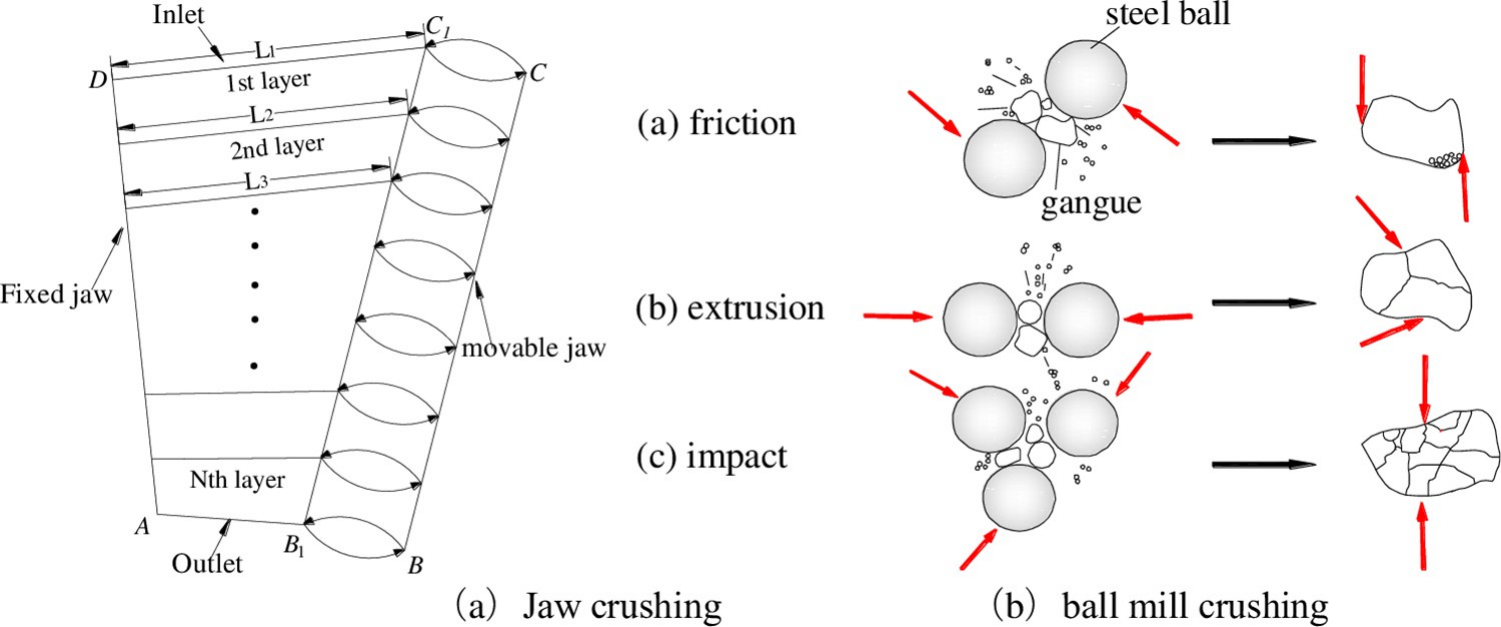
Particle crushing method in the process of “jaw crusher-ball milling”. (a) Jaw crushing. (b) Ball mill crushing.

Considering fractal dimensions from the perspective of crushing form, Fig 13 illustrates the crushing form of gangue in the "jaw crushing-ball milling" process. Since the jaw crusher mainly relies on the extrusion between the movable jaw and the static jaw to crush the rock as depicted in Fig 13(A) [44, 45]. According to the laminated crushing theory, the gangue must go through *n* times of extrusion crushing from the feeding port to the ore discharge port. This leads to the fact that most of the gangue is broken along the defects of its structure during the jaw crushing process, resulting in more angular particles. The smaller the discharge opening, the smaller the particle size and the larger the particle size fractal dimension. However, the particles with prominent edges and corners or with weak skeleton structures are broken and refined many times, and their particle size structure is further complicated. This makes the particle size predictable, but the final particle shape is not fixed. This also leads to the fact that the fractal dimension of the particle size in the jaw-crushing stage is linear, while the shape fractal dimension is unpredictable.

In the process of ball milling, gangue particle size is continuously reduced by the three interaction effects of impact, extrusion and friction with the grinding medium [46, 47]. While in ball milling, as the particle size decreases, the fractal dimension of the particle size increases, and the shape dimension also increases gradually with the continuous grinding. The phenomenon of agglomeration is that after the particles are small to a certain extent, the small particles are squeezed and impacted together under the three functional mechanisms. It makes the original small particles combine into large particles under the action of three mechanisms, causing the average particle size to increase rather than decrease with time. However, the shape of the particles was still maintained in an elliptical state under the electron microscope indicating that the shape dimension was hardly affected.

## 5 Conclusions

Based on the particle size and shape fractal theory, supplemented by laser particle size analyzer, SEM and other mesoscopic research methods. This paper systematically analyzes the particle size and shape fractal characteristics of gangue in the process of "jaw crushing-ball milling". The following conclusions are drawn:

1. The gangue with different grinding particle sizes has shown obvious fractal characteristics in the ball milling process, and the fractal dimension of the particle size decreases with the increase of the grinding particle size, and increases with the increase of the ball milling time. After the agglomeration phenomenon, the fractal dimension decreased with the increase of ball milling time. As the grinding particle size increases, the fractal dimension coefficient of shape increases. By reducing the grinding particle size, the particle shape fractal dimension will be significantly reduced in ball milling. With increasing time, the shape fractal dimension decreases rapidly from 0 to 30 minutes, and from 30 to 60 minutes, the decreasing range gradually slows down. On the basis of this study, we will further study how to select the crushing process parameters (initial particle size, ball-to-powder ratio, rotational speed, ball milling time) for industrial gangue crushing to ensure the least energy consumption and the optimal particle state.
2. Ball-to-powder ratio has a great influence on the shape and particle size fractal dimension of the gangue in the ball milling process. The fractal dimension of gangue particle size increases with time. As the fractal dimension increases, particle distribution becomes more concentrated. The dimension value of particle size is concentrated in the range of 2.5~2.8. The fractal dimension value of shape decreases with time under each spherulite ratio, and the shape of particles gradually develops to spherical and ellipsoid. The range of the final shape dimension is concentrated between 1.06 and 1.16.
3. Taking into account the changes in particle size fractal dimension and shape fractal dimension, and in order to account for grinding effect and grinding energy consumption, it has been determined that in the "jaw crusher-ball mill" crushing process, the in-grinding particle size should be selected from the gangue of 6mm or 8mm discharge opening width, with a ball-to-powder ratio between 6 and 9. The shape fractal dimension value is up to 1.26, and the particle size fractal dimension value is up to 2.80.
4. In the "jaw crushing-ball milling" process, the particle size and shape fractal dimension values of gangue have the characteristics of periodic changes. In the jaw crushing stage, the fractal dimension of particle size shows an increasing trend with the increase of the discharge opening width, and the correlation between particle size and shape fractal dimension is low. Prior to agglomeration in the ball milling, as particle size decreases, the fractal dimension of the particle size increases, and the shape dimension decreases. After the agglomeration phenomenon, the fractal dimension values of shape and particle size showed a downward trend. According to the analysis, the change of the gangue crushing form in the "jaw crushing-ball milling" process is the main driving factor that causes the evolution of the size fractal dimension and the shape fractal dimension.
